# Downregulation of *BANCR* Promotes Aggressiveness in Papillary Thyroid Cancer via the MAPK and PI3K Pathways: Erratum

**DOI:** 10.7150/jca.91256

**Published:** 2023-11-09

**Authors:** Jinjun Zhang, Yaying Du, Xiaoxue Zhang, Mengchen Li, Xingrui Li

**Affiliations:** 1 Department of Thyroid and Breast Surgery, Tongji Hospital, Tongji Medical College of Huazhong University of Science and Technology, Wuhan, Hubei 430030, P.R. China; 2 Department of Obstetrics and Gynecology, Tongji Hospital, Tongji Medical College, Huazhong University of Science and Technology, Wuhan, Hubei 430030, P.R. China

In the original version of our article, there was an error in Figure 2E. Specifically, the representative images of TPC-1 cell line were incorrect. The correct image is provided below. This correction will not affect the results and conclusions. The authors apologize for any inconvenience this may have caused.

## Figures and Tables

**Figure 2:**
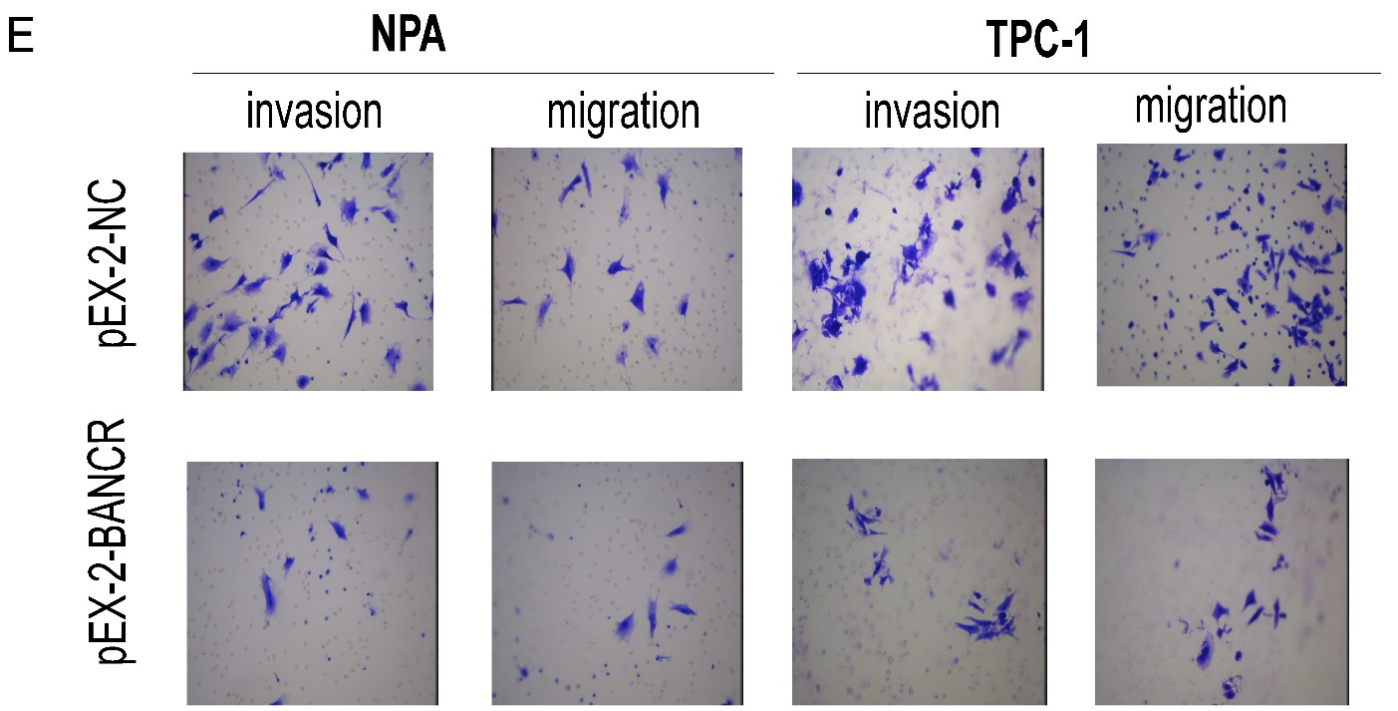
** Effects of *BANCR* on papillary thyroid cancer cell proliferation and motility. (**E) Transwell migration and invasion assays showed that knocking down *BANCR* promoted NPA and TPC1 cell motility. However, cell motility was reduced in *BANCR* -overexpressing NPA and TPC1 cells (crystal violet stain; magnification, ×200). pEX-2-*BANCR*, pEX-2-NC respective plasmid containing human full length *BANCR* cDNA and negative control; *BANCR*, *BRAF*-activated non-protein coding RNA.

